# Kinematic-Based Classification of Social Gestures and Grasping by Humans and Machine Learning Techniques

**DOI:** 10.3389/frobt.2021.699505

**Published:** 2021-10-15

**Authors:** Paul Hemeren, Peter Veto, Serge Thill, Cai Li, Jiong Sun

**Affiliations:** ^1^ School of Informatics, University of Skövde, Skövde, Sweden; ^2^ Donders Institute for Brain, Cognition, and Behaviour, Radboud University, Nijmegen, Netherlands; ^3^ Pin An Technology Co. Ltd., Shenzhen, China; ^4^ Volvo Cars, Göteborg, Sweden

**Keywords:** gesture recognition, social gestures, machine learning, biological motion, kinematics, social signal processing, affective motion

## Abstract

The affective motion of humans conveys messages that other humans perceive and understand without conventional linguistic processing. This ability to classify human movement into meaningful gestures or segments plays also a critical role in creating social interaction between humans and robots. In the research presented here, grasping and social gesture recognition by humans and four machine learning techniques (k-Nearest Neighbor, Locality-Sensitive Hashing Forest, Random Forest and Support Vector Machine) is assessed by using human classification data as a reference for evaluating the classification performance of machine learning techniques for thirty hand/arm gestures. The gestures are rated according to the extent of grasping motion on one task and the extent to which the same gestures are perceived as social according to another task. The results indicate that humans clearly rate differently according to the two different tasks. The machine learning techniques provide a similar classification of the actions according to grasping kinematics and social quality. Furthermore, there is a strong association between gesture kinematics and judgments of grasping and the social quality of the hand/arm gestures. Our results support previous research on intention-from-movement understanding that demonstrates the reliance on kinematic information for perceiving the social aspects and intentions in different grasping actions as well as communicative point-light actions.

## 1 Introduction

In many contexts, social competence relies on successful human-human interaction where people have the ability to recognize and understand human social gestures (hand/arm actions) and transitive gestures that convey intentions when interacting with objects (e.g., McNeill, 1992). Within the area of human-robot interaction (HRI), there is a continuing development of robots to demonstrate relevant social behavior understanding (Breazeal, 2004; Carter et al., 2014; Yang, et al., 2007; Dautenhahn, 2007; Dautenhahn and Saunders, 2011; Kanda and Ishiguro, 2013). This appears to be the case even in industrial settings (Gleeson et al., 2013; Liu and Wang, 2018) as well as in the assisting services and healthcare areas (Cao et al., 2019). The extent to which robots will need to demonstrate this social competence likely depends on the context in which they are used (Fong et al., 2003). From this perspective, the social content of gestures (e.g., McNeill, 1992; Buxbaum et al., 2007), can be based on previous experience of human-human interaction in different contexts. Previous results from (Hemeren and Thill, 2011) demonstrated also an association between the contextual activation of an action representation due to previous experience and the kinematics of the specific grasping action.

In the context of social robotics, gestures are one potentially critical aspect of non-linguistic social interaction (Tversky, 2019) where both the robot and human monitor and influence one another (Lohse, 2011). Movement kinematics have also been shown to be an important source of information (e.g., Ansuini et al., 2014; Becchio et al., 2014; Sciutti et al., 2015). To further investigate this context, human performance and four machine learning (ML) techniques will rate the same kinematically presented gestures according to the level of grasping behavior and according to the level of social behavior. The rating task data from humans are then used as the reference point for determining the quality of ML techniques for classifying gestures into grasping and non-grasping actions as well as social and non-social actions.

In relation to the investigation of grasping and social gestures in this research, Pollick et al. (2001) demonstrated the importance of kinematic variables for affect classification in arm movements by using point-light recordings. Human categorization judgments were performed according to ten possible affects. The results showed that there was considerable variation in the ability of people to identify the affects in the different point-light movements. However, according to two main dimensions in the circumplex model, degree of activation and pleasantness, the correlations between kinematic variables (velocity, acceleration and jerk) and the psychological space resulting from affect classification were very strong. This shows a clear motivation for the use of point-light displays for isolating kinematic variables. In addition, the movement kinematics had a very strong effect on the organization of the psychological space for the perception of affect in the different actions.

Previous results from computational modelling also point towards the connection between kinematic variables and different kinds of gestures and actions. Yu and Lee (2015) investigated the performance of a deep neural network model on intention recognition where eight different kinds of motions were used. They used skeletal nodes of one person to obtain the movement parameters that seemed to characterize the different motions. The recognition rate was nearly perfect for seven out of the eight motions, but there were no social gestures or a systematic investigation of gestures. Bernardin et al. (2005) used a sensor fusion approach for recognizing continuous human grasping sequences. They used a 3D model based on the input from a data glove. They then used Hidden Markov Models to successfully classify 14 different kinds of grasping that could be used to interact with different objects. However, they did not investigate social gestures or the identification of specific gestures. See also Sun et al. (2020) for the use of surface electromyography (sEMG) to recognize dynamic gestures.

In contrast to previous studies, the contribution of the experiments in this article uses both grasping and social gestures, while using vision-based ML techniques, which are described in Section 3.1. A further purpose is to make a direct comparison between human classification and the ML techniques based solely on the kinematic features of human gestures. We use a non-image based glove-technique (Zaini et al., 2013) to record hand actions and then use the data to create visual (image-based) stimuli for human participants. The ML techniques use the 3D coordinates recorded from the glove to learn classifications, which is a skeleton model since the coordinates represent the joints of the hand and arm. One critical aspect in the present research concerns an investigation that, under controlled circumstances, will be able to show whether or not, given the controlled limitations of the study, any performance similarity between human judgments and ML technique judgments can be demonstrated for grasping and social gestures using kinematic (point-light) stimuli.

For the experiments in this research, we created a gesture library with different gesture categories based on previous research. One important contribution of the first experiment was to validate the gesture category exemplars in the library by letting humans judge the degree to which the exemplars belong to the broad categories of grasping, non-grasping, social and non-social gestures.

In order to create kinematic displays of gestures using the hand and arm, we first collected a library of the 3D coordinates of different points on the hand in 105 different gestures. These coordinates were then used to create point-light displays of the different gestures. The kinematics (and spatial position) of the fingers should clearly distinguish grasping from non-grasping, which should also be a sufficient basis for ML techniques to match human judgments (Manera, et al., 2011). For the distinction between social and non-social gestures, the kinematic differences are not as clearly identifiable. The visual distinction between social and non-social gestures might be more dependent upon the previous motor experience of performing the actions in association with a social context (Amoruso and Urgesi, 2016). In this case, classification is a result of shared motor knowledge that integrates perception and action (Sadeghipour and Kopp, 2011). The consequence of this potential dependency is that some ML techniques (e.g., k-Nearest Neighbor) lack a motor repertoire and therefore are perhaps less likely to classify certain kinematic patterns as having a high social content. Thus, ML techniques can be expected to be more successful at learning differences between different grasping actions for object manipulation because of the availability of the kinematic information (opponent motion of the fingers) but less successful at distinguishing social from non-social hand/arm gestures based on kinematic data alone.

This hypothesis was tested by selecting a subset of 30 gestures from the library and then instructing human subjects to rate the extent to which a gesture contained a grasping motion and also to rate the extent to which a gesture is perceived as social. We used human classification data and the kinematics from the 30 actions as input to four ML techniques that learned the association between the kinematic Principal Component Analysis (PCA) profiles. A reasonable assumption is that humans judge actions based on kinematic data although the exact underlying mechanisms are not yet known. Given the mapping between the human data and the kinematics, and to the extent that this occurs for social gestures, ML techniques should help us determine in what way the kinematic profiles might be associated with classification behavior. With a few exceptions, the results suggest that ML techniques can demonstrate a strong association between point-light movement kinematics and the human ratings that led to the classification of grasping and social actions.

The main research questions:

Experiment 1:
    • Accuracy—To what extent do judgments of human participants match the ground truth original classification used for the gestures in the library category structure?
  • The second issue concerns the extent to which humans view the perceptual differences between grasping and non-grasping gestures on the one hand and social vs. non-social gestures on the other. It is possible that accuracy is high in relation to the ground truth but the ratings show that the perceptual differences between the categories is small and/or possibly different for grasping and social gestures.
Experiment 2:
    • Do the selected ML techniques produce similar accuracy and rating judgment levels for the grasping and social gesture categories as for humans in Exp. 1?
  • To what extent do the ML-technique results determine the role of the kinematic profiles for classification behavior such that kinematic information can function as a sufficient basis on which to make social judgments of hand/arm gestures?

## 2 Experiment 1—Human Ratings of Grasping and Social Gestures

The purpose of this experiment was to validate the gesture categories (ground truth) by using a rating task in order to then use the data to compare the human performance with the different ML techniques.

### 2.1 Gesture Library Construction

The gesture library^1^ was created to provide kinematic-based stimulus material to studies on biological motion perception of gestures, which can include areas of action simulation (Liepelt et al., 2010), investigating the neural correlates of the observation of hand actions (Enticott et al., 2010; Streltsova et al., 2010), action segmentation (Hemeren and Thill, 2011) and the design of cognitive systems that interact with humans (Liu and Wang, 2018).

The theoretical and empirical basis for the categorical structure of the gesture library was based on the findings of (Klatzky et al., 1989). Hand gestures that interact with different objects have different kinematic features that also contribute to the creation of motor representations in human cognition. Klatzky et al. (1989) suggest that the cognitive/motoric representation of the hand can be used to model the kind of action (kinematic pattern) that can be used on different kinds of objects. The gestures chosen for the library therefore represent different categorical kinematic patterns, and if the previous results from Klatzky et al. (1989) hold, then results from human ratings in the current experimental conditions should be consistent with those results, which showed that participants made consistent distinctions between grasping and non-grasping (See also Klatzky et al., 1993.). As a further confirmation of the decisive role of the kinematic patterns in gesture recognition, the ML techniques should lead to results similar to the human results.

The gestures are sorted into prehensile and non-prehensile actions according to the different handshape categories proposed by Klatzky et al. (1989) (Table 1). Prehensile actions are further divided into two groups according to the type of grasp used, with precision grip for the pinch category and power grip for the clench category. Similarly, non-prehensile actions can belong to either the palm or the poke subgroup (Klatzky et al., 1989). The category of social gestures in Table 1 consists of hand gestures with communicative content. The list of the gestures in Klatzky et al. (1989) was used as a basis to create some of the social gestures in the library. We then created an additional number of social gestures, which were to be validated by the human rating experiment described below.

**TABLE 1:** Library of gestures grouped by hand action category. Gestures rated in the experiment are in bold and social gestures have a^a^.

Prehensile (grasping)		Non-prehensile (non-grasping)	
Clench	Pinch	Palm	Poke
- bounce small ball	- **cut with scissors**	- **clap**	- attention^a^
- close a jar	- bow with hat^a^	- clap own shoulder^a^	- clean a jar with finger
- close water tap	- deal cards	- clean an apple	- draw with finger
- cut with knife	- **drink from mug**	- clean table	- **feeling fingertips**
- **cut with saw**	- fine^a^	- clean window	- **go over there** ^a^
- **drink from glass**	- open drawers	- come closer^a^	- I’m watching you^a^
- eat apple	- open suitcase	- come in^a^	- **measure distance with**
- flex muscle^a^	- **peel a banana**	- **count 1–5** **^a^**	**fingers**
- hammer	- pick up a pen	- enough^a^	- no-no^a^
- **juggle**	- plug	- flick with hand^a^	- **poke a shoulder** ^a^
- knock on door^a^	- **pull light-cord**	- **give me** **^a^**	- psst^a^
- lift a dumbbell	- put on a cap	- greeting^a^	- quote^a^
- **lift suitcase**	- screw pen	- **high five** **^a^**	- rock-paper-scissors: scissors^a^
- make paper ball and throw	- **tear tape**	- I cannot hear you^a^	- **scratch head** ^a^
- open a door	- tear off page	- impatient fingers^a^	- scratch leg
- **open a jar**	- throw darts	- pat a shoulder^a^	- type
- **open can with opener**	- **unscrew a bottle top and close back**	- **play bongos**	- **use calculator**
- open soda can		- **push with palm**	- thumb up^a^
- open water tap	- unscrew a bottle top	- reach under and lift	
- pour from saltshaker	drink and close back	**- rock-paper-scissors: paper** **^a^**	
- push stapler	- unscrew a bottle top	- roll a carpet	
- rock-paper-scissors: rock^a^	- use door key	- rub stomach^a^	
- sandpaper	- write on board	- salute^a^	
- shake bottle	- zip	- **shake off water**	
- **shake hand** ^a^		- slap^a^	
- snap fingers^a^		- **smooth bedspread**	
- squeeze cloth		- so-so^a^	
- table tennis		- stand-up	
- throw and catch a ball		- stop^a^	
		- stroke a dog	
		- that’s nothing^a^	
		- thinkative^a^	
		- **waving** **^a^**	
		- **what?** **^a^**	
		- whisper^a^	
		- voila^a^	

a The marked actions also belong to the social category.

**FIGURE 1:**
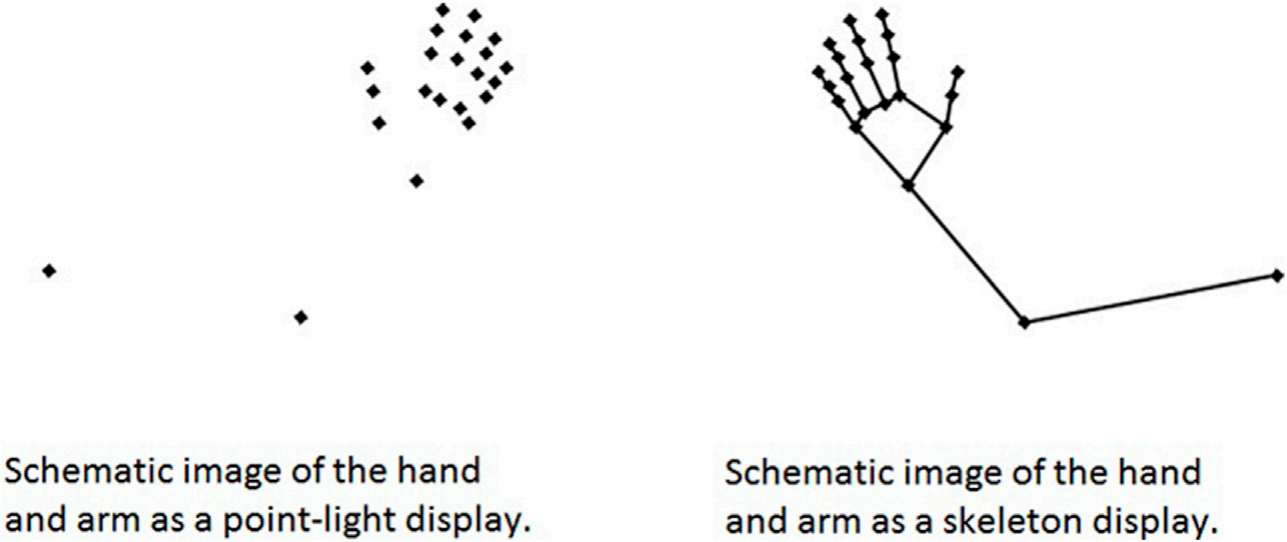
Point-light and skeleton versions of the gesture stimuli.

The recorded 105 gestures are high-resolution sequences of hand and arm movements where the details of the fingers and the hand are not occluded by any rotation or interaction with objects. The movement kinematics are clearly visible. Figure 1 shows a skeleton version and a point-light version. All gestures were presented as point-light displays in the current study.

The library contains original .c3d files with raw three-dimensional coordinates of 34 markers recorded at 60 Hz. The actions were recorded by a Measurand ShapeHandPlus™ and ShapeTape™ motion capture device, with fiber optic sensors capturing trunk position, arm kinematics and the precise movements of the right hand. This recording technique produced a complete three-dimensional representation of the movement of the right shoulder, right arm, hand and fingers for the gestures.

Film recordings (AVI format) of the gestures were then created using the 3D coordinates of 22 of the 34 markers. These markers were depicted as dots to render point-light displays from the same three angles for each action (−45° for a left-frontal view, −135° for a right-frontal view and −180° for a perpendicular right side view). All viewpoints were set to a 10° angle of pitch, presenting a natural sight from slightly above. The film library consists of all actions from all mentioned viewpoints as point-light displays and in a version with the 22 markers connected, forming the skeleton model of the hand and arm. The 22 markers are drawn as white on a black background, with the frame of the display adjusted to the scale of motion in each action. However, with the use of the script in the library, enclosed videos can be easily created from the .c3d files, applying any arbitrary angle and different settings regarding the characteristics of the frame and the model. It is also possible to play the actions directly from the .c3d data.

A right-handed male, one person, performed all of the actions (with the exception of the play bongos and juggle actions) with the right hand, starting and ending them at the same resting position with the arm and hand relaxed at the side. Most of the prehensile actions (i.e. in which the object is held by the hand) also include the movement of lifting up the object before and placing it back to the same place after the action. This method is consistent with Zaini et al. (2013) who also created a library of communicative and non-communicative point-light actions. In another previous study, Yu and Lee (2015) used one person to record motions that were recognized by using a deep dynamic neural model. Results from previous studies suggest that visual discrimination between different action categories is maintained across the kinematic variation that can occur with different people performing the different actions. This result has also been experimentally demonstrated in Alaerts et al. (2011). A central positive consequence of the current study is to demonstrate empirically validated results where human and ML techniques produce similar classifications of gestures.

### 2.2 Materials and Methods

#### 2.2.1 Participants

Forty-eight undergraduate students (24 males and 24 females; 24.6 ± 6.7 years; 4 left- and 44 right-handed) took part in the experiment. Procedures conformed to the Declaration of Helsinki and were previously in a similar experiment approved by the Regional Ethical Review Board of Sweden. Written informed consent was obtained from each participant. Forty-seven participants had a normal or corrected-to-normal vision; one participant indicated uncorrected vision, but was nevertheless able to perceive the figures well. The participants were recruited from two different courses when the lectures were finished, and interested students were asked to simply stay for an additional 40 min in order to participate in the experiment.

#### 2.2.2 Stimuli and Apparatus

The stimuli consisted of thirty gestures quasi-randomly selected from the library in order to achieve a balanced validation of the 105 gestures. Due to the fact that it would take far too long, about 2 hours, for each participant to judge all of the 105 gestures, a representative subset was selected in order to create an acceptable participation time for the participants. The representative subset of gestures was selected according to the extent to which they displayed grasping and the extent to which they could be judged as social (Table 1). Twelve prehensile gestures (6 clenches and 6 pinches), twelve non-prehensile (6 palms and 6 pokes), and 6 additional social gestures were selected in order to reach a total of thirty gestures. This distribution did not, however, create a complete balance between the different categories because the focus on social gestures in this experiment is on the kinematics not on object interaction, which consequently led to more non-grasping than grasping gestures. For the grasping categories, there were 13 grasping and 17 non-grasping gestures, and for the social categories, there were 10 social and 20 non-social gestures.

Video animations were created in MATLAB (Mathworks, Natick, MA) using the 3D coordinates of 22 reference points (representing the shoulder, the elbow, the wrist, each metacarpophalangeal and interphalangeal joint, and the fingertips) from three viewing angles (left-frontal, right-frontal, and perpendicular right side views) for each gesture. The duration of the video animations are presented in Table 2. The 22 reference points are visible in Figure 1. Each gesture was presented from all three viewing angles in order to avoid the situation where only one viewpoint might be more visually advantageous for one gesture compared to another gesture. The order of viewing angles for each action in each trial was left-frontal, right-frontal and then a perpendicular right side view.

**TABLE 2:** Gestures in the experiment. Means and standard deviations (in brackets) of observer ratings for each point-light gesture. N = no and Y = yes, grouped according the labels Prehensile? and Social?

Name of action	Prehensile?		Social?		Duration (s)
	Ground truth	Observer rating	Ground truth	Observer rating	
Clap	N	1.56 (1.21)	N	5.75 (1.13)	8.6
Count 1–5	N	1 (0)	Y	3.6 (2.06)	9.3
Cut with saw	Y	6.25 (0.68)	N	1.38 (0.87)	19.0
Cut with scissors	Y	6.19 (0.91)	N	1.73 (0.96)	19.9
Drink from glass	Y	6.81 (0.75)	N	2.13 (1.67)	13.4
Drink from mug	Y	6.81 (0.54)	N	2.19 (1.64)	12.9
Feeling the fingertips	N	4.75 (1.98)	N	1.69 (1.01)	9.7
Give me	N	2.19 (2.07)	Y	5.47 (1.64)	5.8
Go over there	N	1.69 (1.89)	Y	6.19 (1.05)	7.2
High five	N	1 (0)	Y	5.31 (1.92)	14.6
Juggle	Y	2.88 (2.09)	N	2.85 (1.77)	13.2
Lift suitcase	Y	6.63 (0.89)	N	1.6 (1.55)	17.5
Measure distance with fingers	N	2.81 (1.68)	N	2.46 (2.22)	12.2
Open a jar	Y	4.63 (2.45)	N	1.64 (0.81)	13.6
Open can with opener	Y	6.21 (1.12)	N	1.87 (1.77)	20.9
Peel a banana	Y	4.94 (1.57)	N	2.2 (1.55)	26.1
Play bongos	N	1.06 (0.25)	N	3.93 (1.67)	11.7
Poke a shoulder	N	1.31 (0.70)	Y	5.88 (1.78)	5.1
Pull light cord	Y	5.21 (1.63)	N	2.31 (2.36)	8.3
Push with palm	N	2.69 (1.70)	N	3.36 (1.50)	6.1
RPS-Paper	N	1.38 (0.62)	Y	6.4 (1.24)	7.2
Scratch head	N	2.44 (1.86)	Y	2.07 (1.83)	7.8
Shake hand	Y	6.56 (1.50)	Y	6.25 (2.05)	5.9
Shake off water	N	1.13 (0.34)	N	1.44 (0.81)	7.7
Smooth bedspread	N	1.44 (1.03)	N	1.86 (1.17)	9.0
Tear glue tape	Y	5 (1.71)	N	1.71 (1.27)	10.7
Unscrew bottle top and close back	Y	5.53 (1.41)	N	1.47 (0.92)	14.1
Use calculator	N	2.13 (2.07)	N	3.6 (2.16)	9.8
Waving	N	1 (0)	Y	7 (0)	6.7
What	N	1.25 (0.77)	Y	6.1 (1.07)	5.1

**FIGURE 2:**
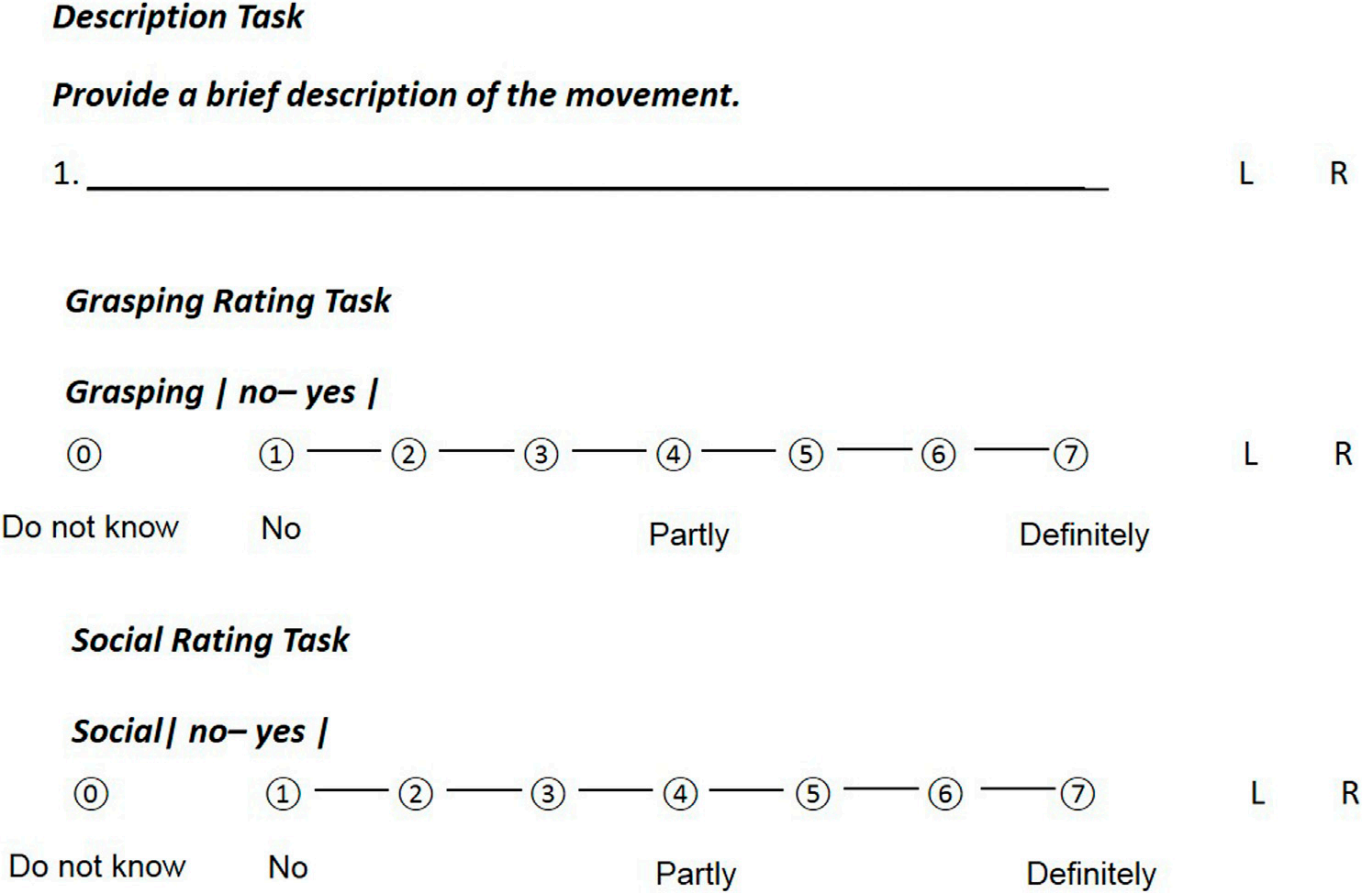
Examples of the three different questionnaire tasks.

Participants were given three different tasks during the presentation of the 30 gestures: 1) provide a short description of the action in the point-light display, 2) rate the extent to which the action represented an instance of grasping and 3) rate the extent to which each action could be perceived as a social action. Ratings were made according to a 7-point Likert scale. Participants were also given the alternative of responding that they were unable to provide an answer. These instances were treated as missing data. See Figure 2 for an example of the three tasks that were given to participants on a paper questionnaire. A further description of the questionnaires is presented in the next section.

A rating task was used instead of a binary classification task to be able to capture data that shows that the different gestures may be perceived as belonging more or less to the different categories. This task is potentially much more realistic than making binary classification judgments. Studies that use binary classification data face greater difficulty in using that data to show graded differences between different gesture examples that belong to the same category.

#### 2.2.3 Design and Procedure

Six different questionnaires (eight participants each) were used. Each questionnaire was used for the 30 gestures. The only difference between the questionnaires was the order of the tasks. This is seen in Table 3. Three of the questionnaires were tested on one occasion in a classroom with 24 participants, while the other three on a different occasion with another 24 participants. All procedures and stimuli were otherwise identical between the two occasions.

**TABLE 3:** Each group of participants viewed the same 30 gestures in the same order, but completed different questionnaires. The questionnaires contained three types of tasks (social, grasping, and description). The tasks were thus presented in a counterbalanced order for each group according to 10 gestures each.

Stimuli gestures	Task questionnaires (Q1-Q6)					
	Q 1	Q 2	Q 3	Q 4	Q 5	Q 6
Gestures 1–10	Social?	Social?	Grasping?	Grasping?	Description	Description
Gestures 11–20	Grasping?	Description	Description	Social?	Social?	Grasping?
Gestures 21–30	Description	Grasping?	Social?	Description	Grasping?	Social?

To avoid influencing one another when participating under the same experimental conditions, participants sitting next to one another were given different questionnaires of the three types mentioned above. This resulted in a design where eight participants, while viewing the same gestures, were individually doing Task 1 while eight participants were individually doing Task 2 and eight participants were individually doing Task 3. Participants were informed that they would be performing different tasks and therefore could not assist one another while viewing the same gesture. Given this design, each participant viewed the same gestures in the same order but was performing different tasks. The counter-balanced order of the three tasks is presented in Table 3.

Due to this counter-balanced task ordering, the order of presented gestures had to remain the same for all questionnaires. The questionnaires are presented as supplementary material as well as the original film sequence of the complete gesture sequence that was used for all participants.

Stimuli were projected on a classroom screen, and responses were recorded via individual questionnaires that were passed out. The experimental session started with an introduction and three training gestures, demonstrating each of the three tasks. Each trial (for both training and experimental gestures) consisted of a set of three consecutive videos, presenting the same gesture from the three viewing angles as described above, always in a fixed order. Participants were informed that they were viewing the same action three times from different viewing angles, and they responded to them in the 20 s provided after the stimulus presentation. Trial numbers were shown at the beginning of each trial to assure that participants wrote their responses to the correct item. The stimulus film with the training and testing gestures was 30 min long. This design ensured that each action was only presented once and thus no carry-over effects could take place from one task to another. Since there were three different tasks and a total of 48 participants, 16 independent responses were obtained for each gesture and task.

#### 2.2.4 Analysis

Descriptive statistics were used to assess the human raters’ perceptual accuracy of each action. Identification accuracy was also measured as an indicator of the ability of the movement kinematics to reliably portray the specific gestures. The grouping of actions based on human ratings was analyzed in a hierarchical cluster analysis both for the grasping and social dimensions. As a measure of inter-rater reliability, the intraclass correlation coefficient (ICC) was used. In our experiment, three different subsets of randomly selected raters assessed three different subsets of actions for each task. While a one-way random model would apply to situations where each item or each subset of items was rated by a different subset of raters, a two-way random model uses the same set of raters. Since the three groups of participants completed different questionnaires and solved the tasks on different subsets of actions and in different orders, we applied a one-way random model rather than a two-way random model (Koo and Li, 2016). Single-rater type ICC estimates and 95% confidence intervals (CI) were calculated, together with all other analyses, in SPSS version 24 (SPSS Inc., Chicago, IL). Statistical t-tests were also used to determine the significance of the differences between the different rating conditions.

Data analyses address two issues. The first concerns the accuracy of the human judgment data in relation to the ground truth presented in Table 2. This analysis addresses the validation of the original classification of the gestures when the gesture library was made. Does the human judgment data confirm the categorical assignments of grasping vs. non-grasping and social vs. non-social actions? The second issue concerns the extent to which humans view the perceptual differences between grasping and non-grasping gestures on the one hand and social vs. non-social gestures on the other. It is possible that accuracy is high in relation to the ground truth but the ratings show that the perceptual differences between the categories are small.

The rating dataset was then used to create dendrograms to visualize the categories according to grasping vs. non-grasping and social vs. non-social. An agglomerative hierarchical cluster analysis in SPSS version 24 (using squared Euclidean distance and within-groups linkage) was used on the raw rating data for each gesture and participant rating to produce the dendrogram (Yim and Ramdeen, 2015). The dendrogram could then illustrate the rating differences between grasping and non-grasping on the one hand and social and non-social ratings on the other.

### 2.3 Results

The raw data showed some missing values. Some participants responded that they were not able to judge the extent of grasping or social content of the gestures, which was counted as missing data. The missing data was 4.7% of all responses; 8.3% in the social task and 1% in the grasp task. The remaining data was used to determine the mean rating for each gesture and is presented in Table 2. For the grasping ratings, any value above 4 indicates that participants perceived the gesture as grasping, and any value below 4 indicates that the gesture was perceived as non-grasping. For social gestures, ratings above 4 indicate a social gesture judgment, and values below 4 indicate that the gesture was seen as non-social.

Regarding the issue of accuracy, confusion matrices in Table 4 show a few errors but also that human judgments quite clearly agree with the ground truth initial classifications for both grasping and social gestures. The errors for the grasping gesture ratings were for “feeling the finger tips” where ratings indicated grasping rather than non-grasping and for “juggle” where the ratings indicated non-grasping rather than grasping. For the social vs. non-social ratings, participants viewed “clap” as social rather than non-social, which indicates that the original classification might have been erroneous. The other two errors indicate that participants rated initially determined social gestures as non-social. Both gestures “count 1–5” and “scratch head” were rated as non-social in relation to the ground truth value of being social gestures.

**TABLE 4:** Confusion matrices for human judgments of gestures as a function of judgment task (grasping and social).

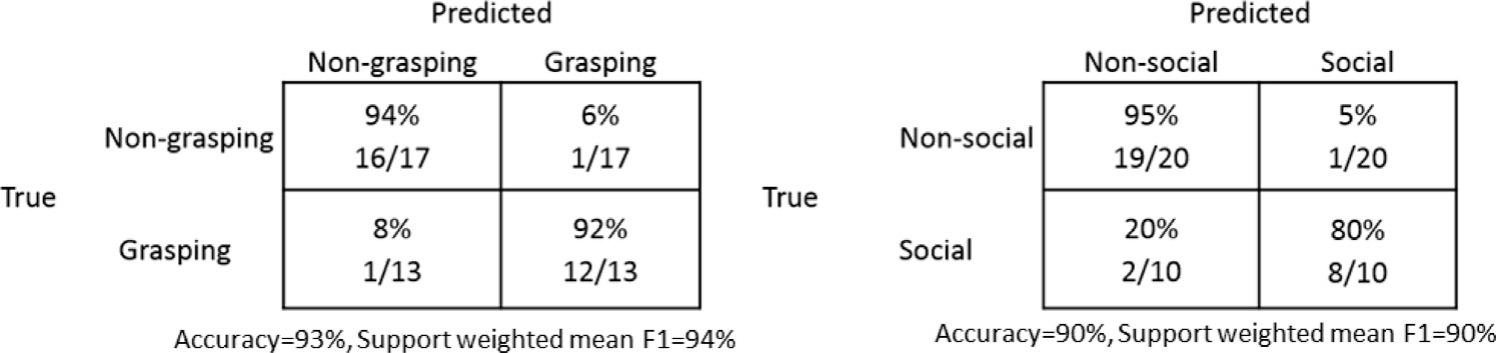

**FIGURE 3:**
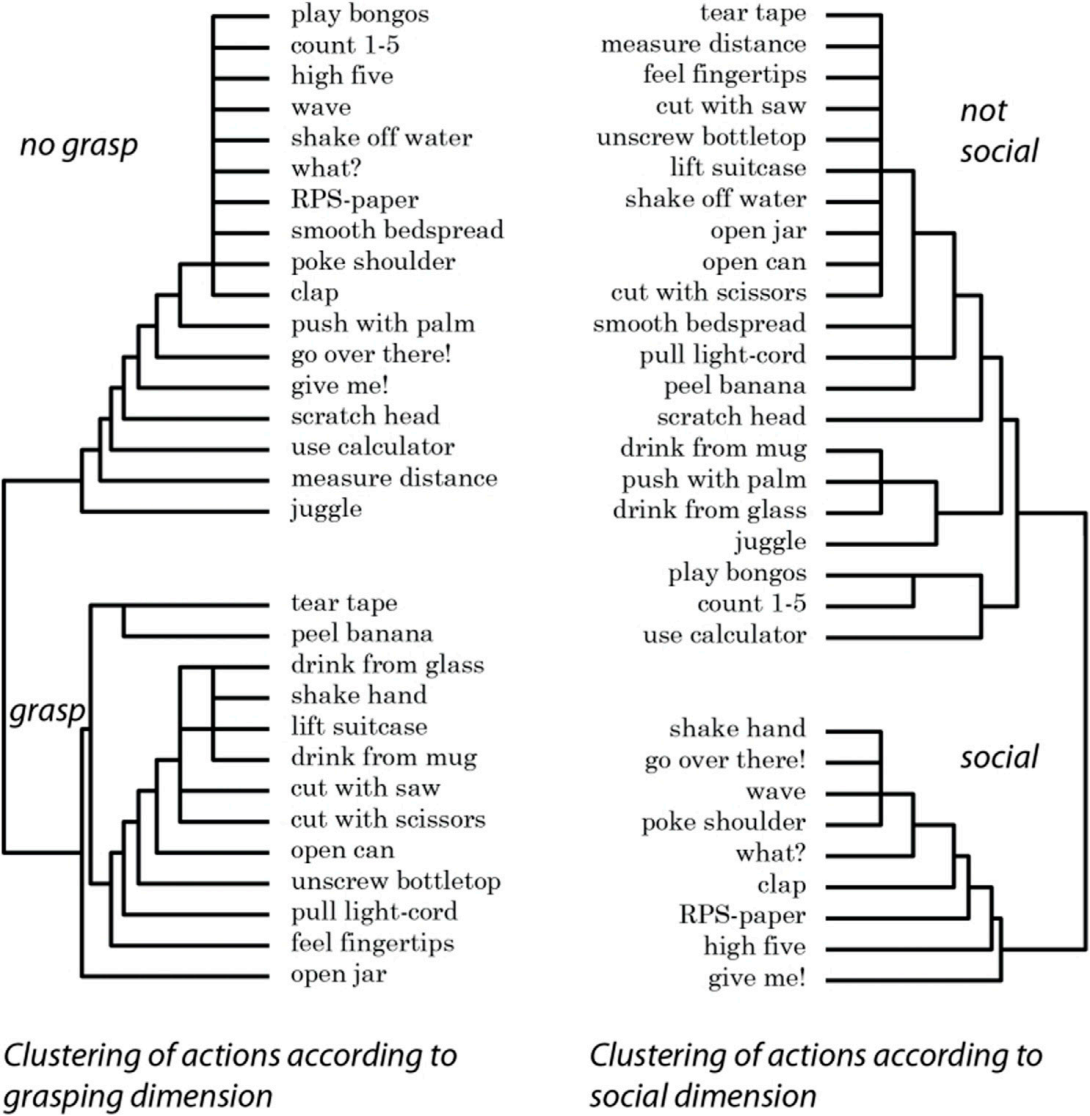
Clustering of gestures as a function of participant ratings of the degree of grasping and the extent to which the gestures were social.

The dendrograms are presented in Figure 3. The major clusters show that the participants perceived the same gestures differently depending on the categories (grasping vs. non-grasping and social vs. non-social) used to rate the gestures. The clear distances between the major clusters also indicate the extent to which people visually discriminate between the gestures according to the studied categories. In order to further quantitatively test the extent to which people view these perceptual differences, the difference between the means for the two different cluster pairs was significant. The mean rating for the thirteen gestures in the grasping cluster was 5.81 (sd = 0.82), and for the non-grasping cluster (n = 17) the mean was 1.70 (sd = 0.67), *t*(28) = 15.01, *p* < 0.001, 95% difference CI (3.55, 4.67). The mean rating for the 9 gestures in the social cluster was 5.53 (sd = 1.56), and for the non-social cluster (n = 21) it was 2.46 (sd = 1.15), *t*(28) = 6.03, *p* < 0.001, 95% difference CI (2.02, 4.11])

Regarding the potential difference between the mean judgment ratings for non-grasping and non-social gestures (1.70 vs. 2.46), there was no significant difference, *t*(16) = 1.56, *p* = 0.138. There was also no significant difference between the mean judgment ratings for grasping and social gestures (5.81 vs. 5.53), *t*(9) = 0.34, *p* = 0.746.

Single-measure ICCs for the grasping [0.729, 95% CI (0.625, 0.840)] and the social ratings [0.700, 95% CI (0.506, 0.889)] dimensions indicate a moderate to excellent agreement (Cicchetti, 1994) between test participants in both tasks. These values were calculated after a listwise exclusion of participants with missing values (4 and 20 participants, respectively; see also section 3.2.3) and showed a high level of significance, *F*(25,39) = 44.07, *p* < 0.001; and *F*(9,150) = 38.30, *p* < 0.001, respectively.

### 2.4 Discussion

These results show that humans seem to make clear judgments between grasping and non-grasping gestures on the one hand and social and non-social gestures on the other. This occurs when they are given the different judgment tasks for the same stimuli. An explanation for the different ratings for the grasping judgments is that people are using the kinematic information in the high-resolution point-light displays to track the motion of the fingers in relation to hand and arm movement. As the hand and arm move, the fingers may also be moving to prepare for an interaction with an object. Indeed, the different motion and position patterns of the fingers and hand are factors that seem to produce different visual patterns that define different kinds of grasping. However, when it comes to the social judgments, it may be the case that additional contextual knowledge of human social interaction is needed, i.e., social experience that frames the kinematic information (Amoruso and Urgesi, 2016). For example, understanding the kinematics of a “high five” gesture may require contextual knowledge about how to motorically respond in a social situation as an expression of agreement. It could also be the case that the available kinematic information is sufficient for the ability to distinguish between these two gesture categories.

In order to test this, we submitted the 3D marker data (22 markers) in the original gesture files to PCA to reduce the amount of noise (dimension reduction) in the data and to maximize the amount of variance associated with the most informative component in the original data. The PCA profiles for the different gestures were used as input into four different ML techniques to see if any of them would yield a result similar to the human data. This should not be confused with the task of predicting the human data. The comparison here is to assess whether selected ML techniques will produce similar accuracy and difference results.

## 3 Experiment 2: Machine Ratings of Grasping and Social Gestures

### 3.1 ML Techniques—Materials and Methods

Two fairly recent extensive surveys on gesture recognition have been conducted (Rautaray and Agrawal, 2015; Liu and Wang, 2018) and discuss the current trends in sensor technology and ML techniques. The contexts of the surveys have been in the area of human computer interaction (Rautaray and Agrawal, 2015) and human-robot collaboration (Liu and Wang, 2018). The purpose of the studies presented in this article can contribute to both areas, especially human-robot collaboration by investigating the performance of the four ML techniques with regard to ratings of grasping and social gestures. If ML techniques show similar results to human ratings, especially for social gestures, artificial systems can use the kinematics to detect, recognize and react to human gestures that require social interaction in service settings and in the context of human-robot collaboration in industrial manufacturing.

The classifications here will be based on the similarities of time-series kinematics and verify the corresponding score consistency based on human ratings. PCA will be used to find the principle component (containing the largest variance and most information) in the original 3D data from the glove recordings. This component (position variance), which retains the core descriptive kinematic profiles of each gesture, will be used as the input to the different ML techniques.

We assume that if the classification algorithms can produce ratings that are statistically comparable to human ratings based on the input to the algorithms, then kinematics can be used to recognize social/non-social or grasping/non-grasping actions. We used four classification algorithms k-Nearest Neighbor (kNN; Cover and Hart, 1995), Locality-Sensitive Hashing Forest (LSH-F; Bawa et al., 2005), Random Forest (RF; Breiman, 2001), and Support Vector Machine (SVM; Hearst, 1998; Vapnik, 2013) to achieve our experimental aims. The results indicate that kinematic information can function as a sufficient basis on which to make social judgments of hand/arm gestures. It is important to point out that the ground truth in the current experiment is the original classification of gestures according to Table 2, i.e., not the human performance and rating data to which the algorithms are then compared.

The tools used in our work to implement these ML algorithms are based on Python and the scikit-learn library. The same 30 actions used in the human experiment are used for testing, and 4-fold cross validation (to avoid overfitting because of the limited size of our dataset) is applied to the remaining 75 actions to find the best parameters for the model.

kNN is at the core of many key classification applications. A naïve approach to kNN uses a direct calculation of distance to find the k closest neighbors to determine the class of a target (Marasović and Papić, 2012). The kNN algorithm relies heavily on the training data. For example, if the training data contain too many datasets, on-line distance calculation and neighbor searching might be slowed down drastically. A condensation/reduction preprocessing is thus normally needed to remove outliers or redundant datasets (Bhatia, 2010). In our work, as our datasets are limited, we can skip this step and directly cross-validate to find the optimal k value, which is 9.

Another approach to searching similarities from an immense number of data points is called LSH (Shakhnarovich et al., 2006). Instead of solving the classification problem exactly like kNN, LSH tries to find a set of data points that are approximately the closest to the target. The approximation is due to the fact that perfect hash functions may not exist or are extremely hard to find. LSH-F (Bawa et al., 2005) is an extension of LSH. It orders hash functions in tree structures to randomly classify data into leaves through feature dimensions. The theory behind this is that when data gets pushed to leaves of different trees, similar datasets always generate similar patterns amongst tree leaves. Then a cosine distance can be calculated to search similarities within a large database (Van Durme and Lall, 2010; Leskovec et al., 2014). This approach avoids the possible computational cost of kNN algorithms related to a large amount of data.

The RF algorithm is based on ensemble learning, which joins different or the same types of algorithms multiple times to form a more powerful prediction model. It combines multiple decision trees to form a forest of trees. Data samples are randomly selected to create decision trees. Each tree in the forest outputs a prediction and the best solution is selected by means of voting. The optimal number of trees for our purposes was found to be 9, using the grid search cross-validation. The grid search method tries all possible k-values, for example the integer values from 1 to 50.

An SVM is a discriminative classifier whose objective is to find a hyperplane in an N-dimensional space that separates data points with the largest amount of margin. A hyperplane is a decision boundary that helps to classify a set of objects with different class labels. Each side of the hyperplane belongs to data points of different classes. The kernel of the SVM used in our experiment is polynomial with degree = 3. In order to take into account the imbalanced data, the weight class for grasping gestures was 0.55, for non-grasping 0.45, for social gestures 0.59 and for non-social gestures 0.41.

We use kNN, LSH-F, RF and SVM to classify our profile data because they are state-of-the-art classification methods. The comparisons of classification results with human ratings in the next section show which method is better for classifying human kinematic movement.

#### 3.1.2 Data

The human participants in the experiments rated the extent to which a particular action is either perceived as a social or grasping instance using a 7-point Likert scale. Since the purpose of this research is to compare (not predict) the human ratings with ML-techniques, an additional mapping function is applied to the outputs of ML techniques based on their probabilities (Murphy, 2012) so that they are transformed to the same 7-point Likert scale as human ratings. The probability calculation was the result of a function call from the open source Python library (Scikit-learn). The mapping function can be described as: $R_{ML}=P\left(class|input\right)\times 6+1$ , where P(class|input) is the probability of an input data point belonging to either a grasping or a social action, e.g., if the probability of an action being grasping is 1, the transformed ML rating for this action is $R_{ML}=7$ . The mapping function assumes a linear relationship between the 7-point-likert scale and the probability *P*. For example, value 7 in the scale to has the probability 1 to be in class x, value 1 in the scale for the opposite, and value 4 has equal probability to be in either class. Therefore, a comparison can be made between the human ratings and ML outputs.

For kNN and LSH-F, this probability is calculated as: *P*(*class|* *input*), where $N_{total}$ is the total number of n nearest neighbors and $N_{class}$ is the number of neighbors predicted to be this class. For random forest, the probability is computed as the mean predicted class probabilities of the trees in the forest^2^.

Platt scaling (Platt, 2000) trains the parameters of an additional sigmoid function on top of SVM to map the SVM outputs into probabilities so that the classifier outputs a calibrated posterior probability. It is used to obtain the probability of a given data point belonging to a particular class instead of the distance of that data point to the boundary. Platt scaling optimizes the probability of an input data point belonging to a class by calculating $P\left(class|input\right)\approx \frac{1}{1+\exp\left(A*f\left(input\right)+B\right)}$ , where f(input) is the signed distance of the input data point from the boundary plane. Platt scaling trains a probability model on top of SVM.

To illustrate the comparison between results based on kinematics classification and experiments on human subjects from previous research, our experiments use the following steps:

Step 1. Classification: PCA was applied to the normalized 3D position data of the 22 markers that were used in the human experiment. The data used for representing each gesture is constructed as a matrix with size of *a* by *b*, where *a* is the number of samples (different for each action) and *b* is the number of kinematic points (*b* = 102). Firstly, in order to compare similarities between gestures represented by a 2D Matrix, we chose to reduce the dimensions of the data from 2D to 1D by finding the kinematic points with salient variance during sampling time. PCA (principal component analysis) might be a suitable technique for handling unsupervised dimension reduction. After being processed with PCA, the dataset for each gesture was multiplied with the salient weights and transformed to a 1D dataset with size 1 by *a*. Secondly, since *a* is different for each action, each gesture dataset was reconstructed into datasets with an equal sampling time length by shifting the final values of each compressed dataset repeatedly to the maximum length of sampling time. Each gesture was extended to 1,800 samples, resulting in a feature dimension of 1,800 × 1.
Different ML techniques were then applied to the profile data of PCA output, which is the position variance of the different markers of the hand model according to the different gestures.
Step 2. Projection: Map the classification results to a 7-point Likert scale, which is the same as used in human ratings.
Step 3. Statistical analysis: Compare the ratings from the kNN, LSH-F, Random Forest and SVM with the ratings found in the human data.
Step 4. Visualization: Qualitatively display similarities for different techniques and verify rating consistency comparing different ML rating methods with human ratings for each gesture type.

### 3.2 Results

#### 3.2.1 Classification Performance with Respect to Ground Truth

##### Grasping and Non-grasping Gestures

Table 5 shows the confusion matrices of the classification results from kNN, LSH-F, RF, and SVM. Since we have an uneven class distribution and both false positive and false negative cases are taken into account, the support weighted mean F1 scores are calculated. A high value of an F1 score indicates relatively high values of both precision and recall.

**TABLE 5:** Confusion matrices for classification results for grasping from kNN, LSH-F, RF and SVM.

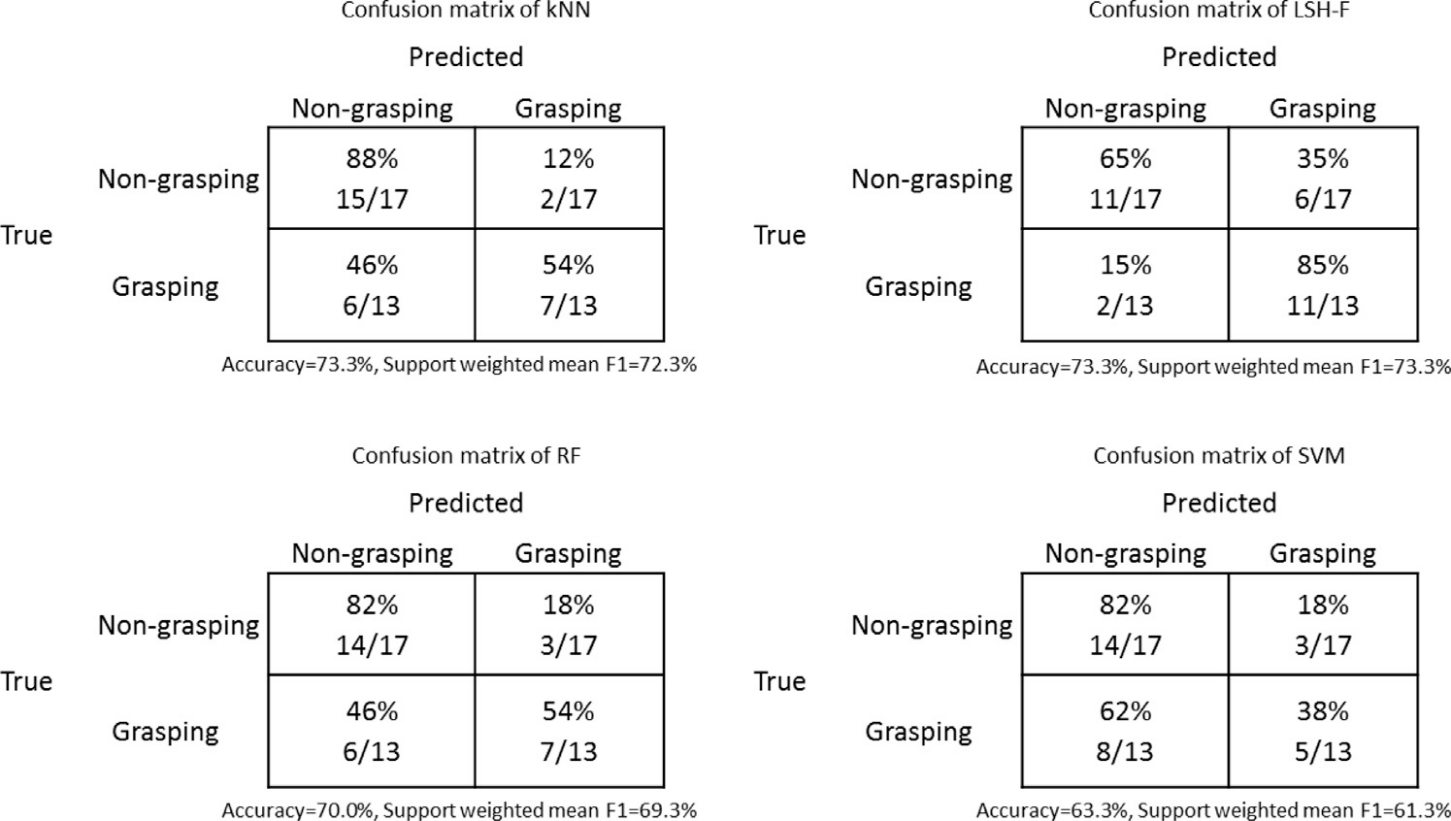

**FIGURE 4:**
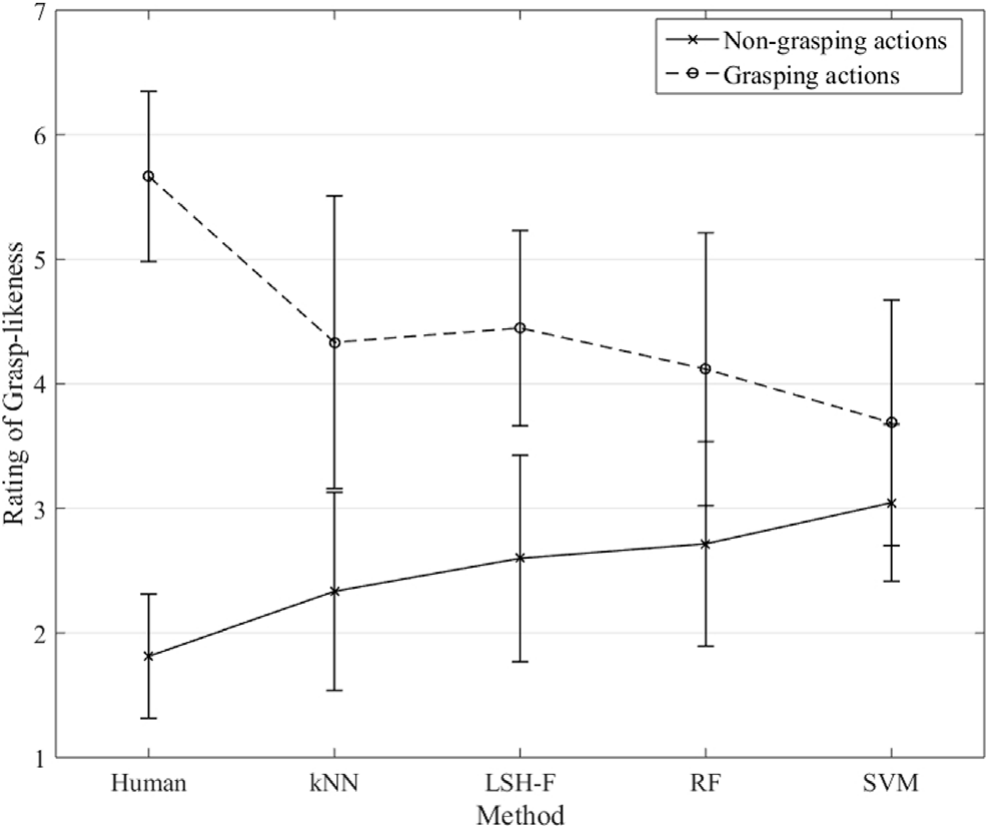
Rating means for grasping and non-grasping gestures for humans and ML techniques (Error bars represent 95% CI.).

The rating means for the different ML techniques and for the human ratings for grasping and non-grasping are illustrated in Figure 4.

A two-way mixed (2 × 5) ANOVA was performed to test the effect of the repeated-measure grasping-gesture category (non-grasping and grasping) in relation to the five different methods (between-groups factor). There was a significant main effect of the grasping-gesture category, *F*(1, 60) = 98.35, *p* < 0.0001, η2 = 0.62. There was no significant main effect of the method factor, *F*(4, 60) < 1. The interaction effect was significant, *F*(4, 60) = 6.57, *p* < 0.001, η2 = 0.31.

Figure 4 shows the interaction pattern. Human ratings showed a clear and large difference between the two grasping gesture categories as described in section 2.3 for the human data. This difference is, however, smaller for the ML techniques. The simple main effect of the non-grasping ratings as a function of the different methods was not significant, *F*(4, 80) = 1.82, *p* = 0.134. The simple main effect of grasping ratings as a function of the different methods, however, was significant, *F*(4, 60) = 2.80, *p* = 0.034, η2 = 0.157.

A further detailed statistical analyses of the paired sample confidence intervals for the different methods as a function of grasping-gesture categories show that the differences between mean ratings for grasping and non-grasping are significant for kNN (95% CI [0.70, 3.30]), significant for LSH-F (95% CI [0.76, 2.94]), significant for Random forest (95% CI [0.13, 2.68]), and not significant for SVM (95% CI [-0.43, 1.71]). The non-significant results for SVM therefore contribute to the significant interaction effect.

Although the differences are not as large as for the human ratings, the ML techniques (with SVM as an exception) appear to reliably create the two categories of grasping and non-grasping actions that can be distinguished on the basis of the kinematic information in the PCA profiles.

##### Social and Non-social Gestures

Table 6 shows the confusion matrices of the classification results from kNN, LSH-F, RF, and SVM. Since there is an uneven class distribution and both false positive and false negative cases are taken into account, the support weighted mean F1 scores are calculated. A high value of an F1 score indicates relatively high values of both precision and recall.

**TABLE 6:** The confusion matrices for the classification results for social gestures from kNN, LSH-F, RF, and SVM.

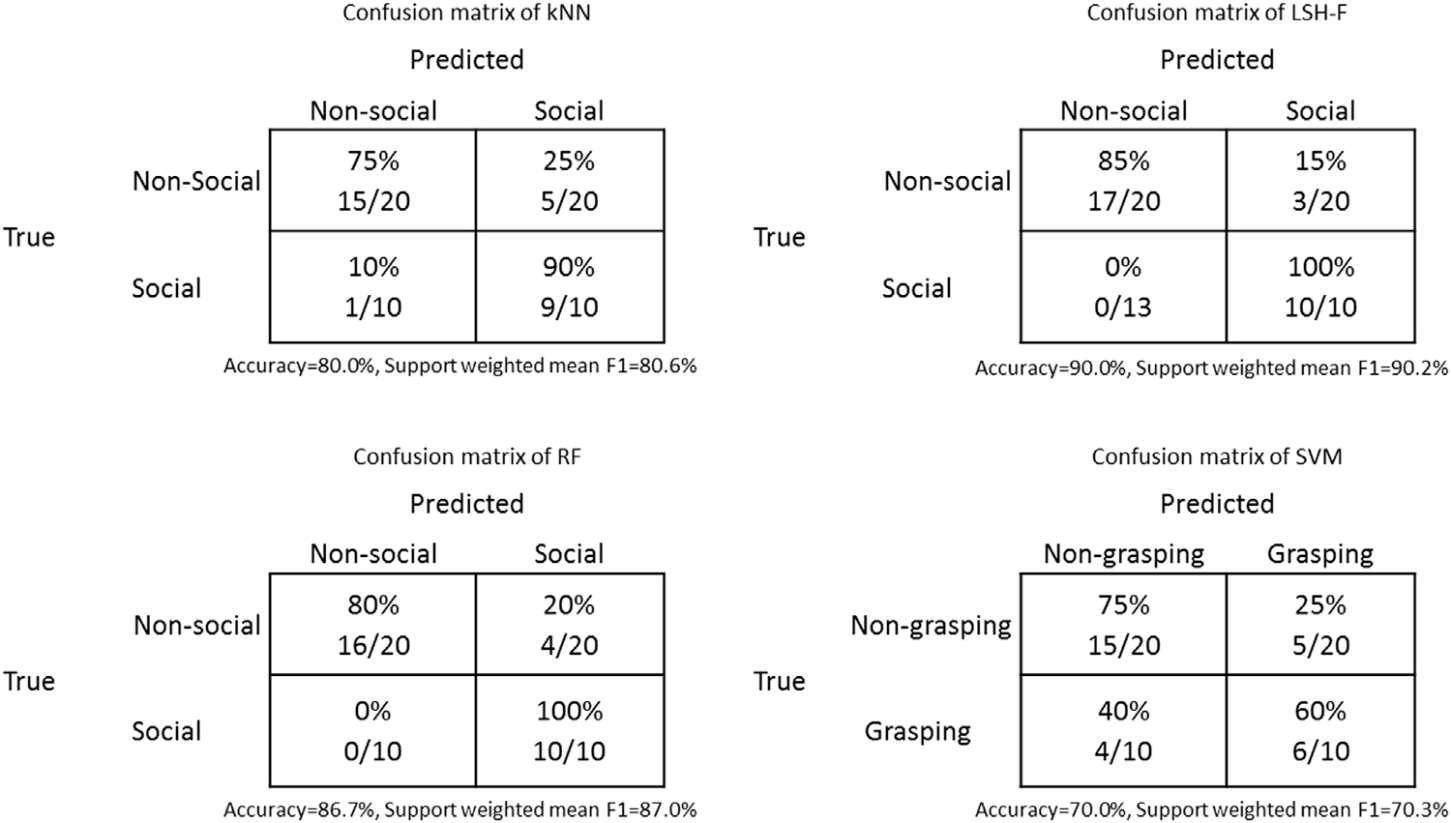

**FIGURE 5:**
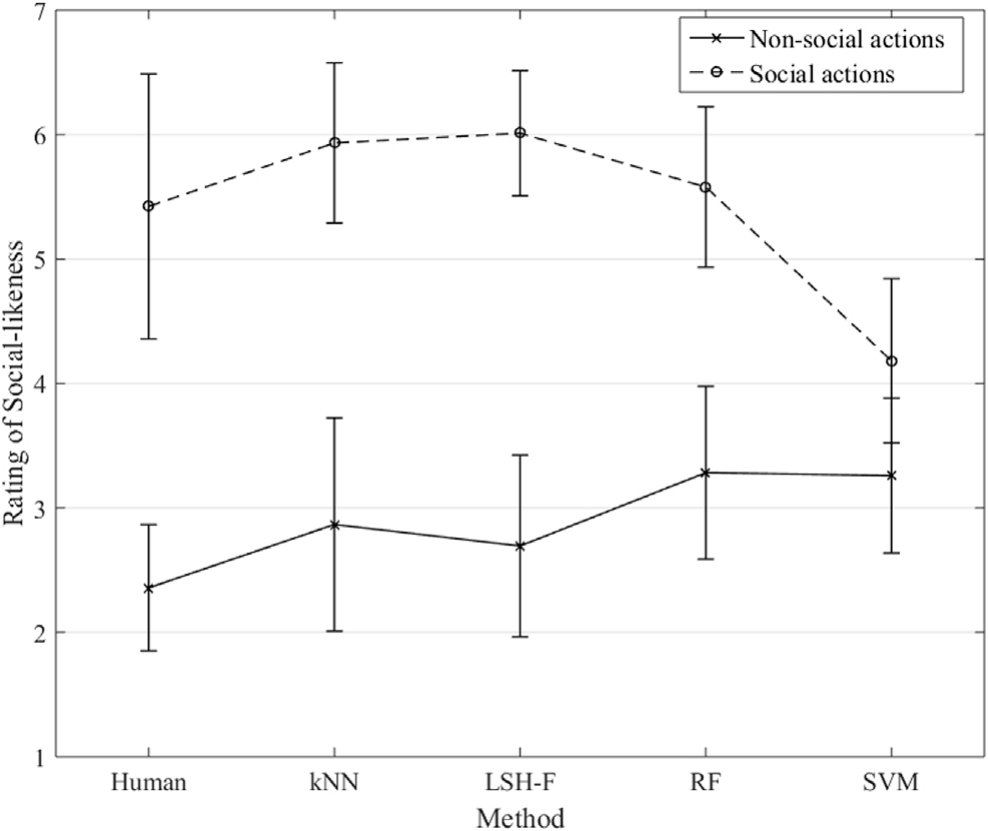
Rating means for non-social and social gestures for humans and ML techniques (Error bars represent 95% CI.).

The rating means for the different ML techniques and for the human ratings for social and non-social categories are illustrated in Figure 5. Here it is clearly the case that the ratings based on ML techniques are very similar to the human ratings, the only difference being for SVM.

Similar to the previous analysis, a two-way mixed (2 × 5) ANOVA was performed to test the effect of the repeated-measure social-gesture category (social and non-social) in relation to the five different methods (between-groups factor). There was a significant main effect of the social-gesture category, *F*(1, 45) = 155.51, *p* < 0.0001, η2 = 0.78. There was no significant main effect of the methods factor, *F*(4, 45) = 1.69, *p* = 0.17, η2 = 0.13 The interaction effect was significant, *F*(4, 45) = 3.56, *p* = 0.013, η2 = 0.24.

The simple main effect of the social gesture rankings, was significant, *F*(4, 45) = 5.24, *p* = 0.001, η2 = 0.32. SVM is significantly lower in its rating of the social gestures compared to the other ML-techniques, SVM vs. kNN, 95% CI [−3.09, −0.41], SVM vs. LSH-F, 95% CI [−3.17, −0.49], and SVM vs. RF, 95% CI [−2.74, −0.053]. The difference between SVM and the human result was, however, not significant, 95% CI [−2.58, 0.10]. There were no significant differences between the human rating mean for the social gestures and the ML techniques, all *p*s > 0.05. For the simple main effect of the non-social gesture ratings, there were no significant differences, *F*(4, 95) = 1.40, *p* = 0.24, η2 = 0.056.

According to Figure 5, the ML techniques appear to reliably create the two categories of social and non-social actions that can be distinguished on the basis of the kinematic information in the PCA profiles, with the exception of SVM.

#### 3.2.2 Comparison to Human Ratings

The purpose of this section is to show some key similarities and differences between human and ML techniques for specific gestures.

**FIGURE 6:**
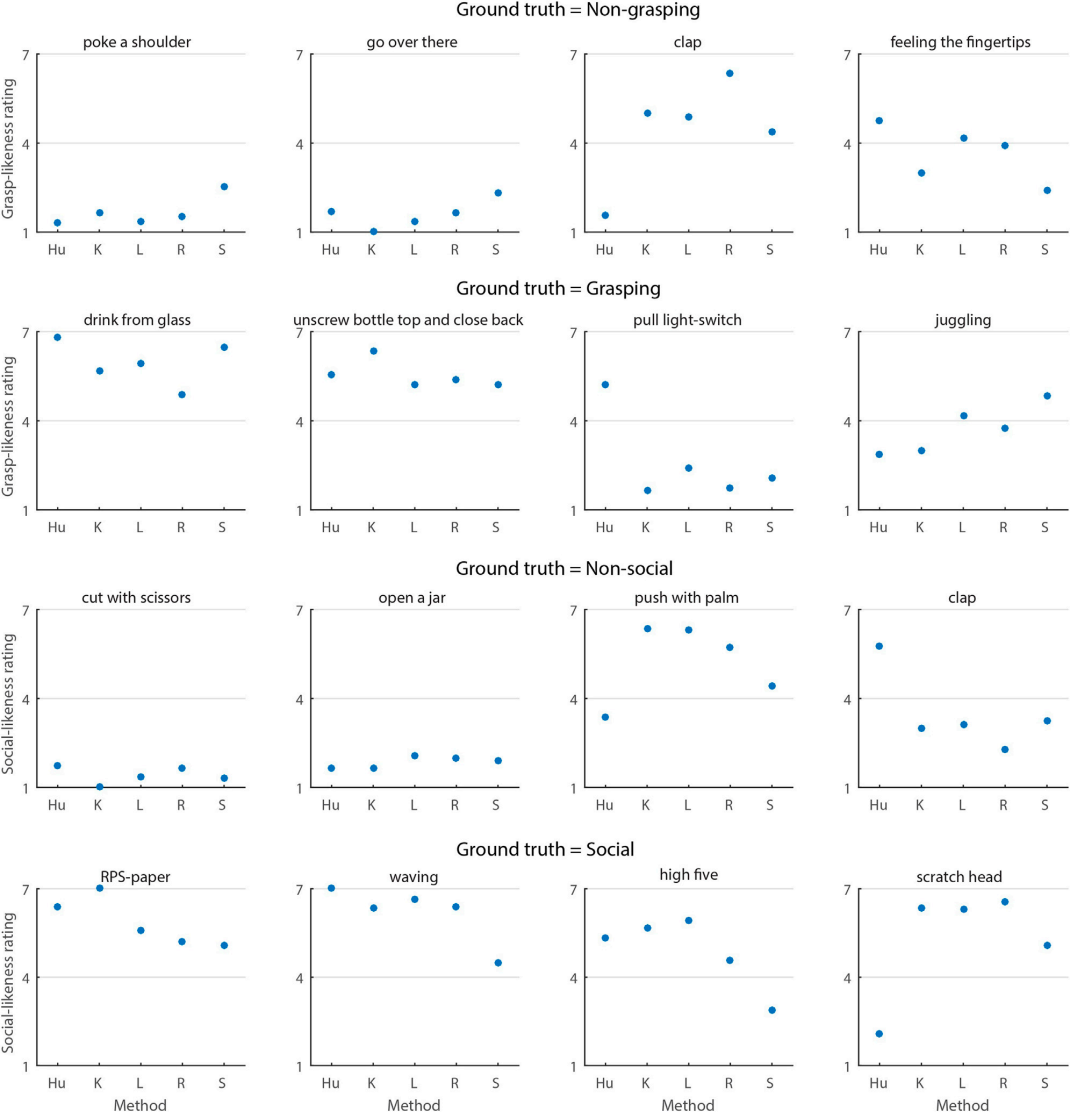
The dotted line in each subplot separates non-grasping **(lower part)** from grasping **(upper part)**, or non-social **(lower part)** from social **(upper part)**. The values on the *y*-axis are ratings from the 7-point Likert scale. The values on the *x*-axis are different methods: Hu = human ratings; K = kNN; L = LSH-F; R = random forest; S = SVM.

In order to qualitatively compare the rating values between human and ML methods and verify the consistency of gesture judgments, the ratings of each gesture from different methods are plotted Figure 6.

Sixteen gestures were selected out of the 30 gestures from the testing dataset. The selected gestures show all cases where the human ratings agreed with ML techniques and the cases where human ratings differed from ML techniques. The complete plots of all 30 gestures can be found in the complementary materials.

The non-grasping group of plots show that human ratings are not always correct: it classified “feeling the fingertips” as a grasping action, while all four ML methods classified it as non-grasping. The kNN, LSH-F and Random forest are quite consistent with human ratings in non-grasping gestures, which is also verified by our pairwise comparisons.

In the grasping group, the human rating is wrong about the gesture “juggle” while LSH-F and SVM are correct. However, all or most of the ML techniques are wrong about “drink from mug”, “pull light cord”, “shake hand” and “tear tape”. In general, the LSH-F is better in consistency than the other three and the SVM performs the worst.

The non-social group plots show that all four ML techniques disagree with human ratings in “clap”, “pull light cord”, “push with palm” and “shake off water”, among which “clap” is judged by humans as a social gesture. kNN, LSH-F and random forest are more consistent with human ratings than SVM in this group according to the means in Figure 5.

All four ML techniques are different from human ratings only in the action “scratch head” in the social group, which is also the only case when humans make a mistake, and all ML methods are correct. Apart from this, the kNN, LSH-F and random forest are quite close to the human ratings. It is previously confirmed by the statistical analyses.

## 4 Discussion and Conclusions

The accuracy results from Experiment 1 demonstrated a clear matching with the ground truth original classification used for the gestures. The rating results also showed that accuracy was an effect of the clear perceptual differences between grasping and non-grasping gestures on the one hand and social vs. non-social gestures on the other. The difference in these rating results suggest a more binary perceptual categorization even for social and non-social gestures, which is similar to categorical perception results for familiar objects (Newell and Bülthoff, 2002).

The results from Experiment 2 showed a somewhat lower accuracy tendency in relation to human performance, but the rating results clearly indicate similar levels of rating differences compared to human performance. The ML techniques, with the exception of SVM, not only demonstrated a clear difference between grasping and non-grasping gestures but also a very clear difference between social and non-social gestures. This result was expected based on results from previous research showing that humans and ML techniques use kinematic information to classify grasping gestures (Zaini et al., 2013; Rautaray and Agrawal, 2015; Cavallo et al., 2016; Liu and Wang, 2018). Grasping is about moving the arm and fingers to interact with an object. The necessary information is in the display.

The ratings of the extent to which a gesture is social have not been previously studied, particularly in relation to a similar task for ML techniques. The major contribution here is a demonstration that there appears to be sufficient kinematic information in the PCA input to allow three of the four ML-techniques to make similar judgments for social gestures as humans.

One important issue in our results concerns the more specific information that contributes to the classification distinction between social and non-social gestures given by the kinematic patterns of hand and arm movement. The input to the ML techniques was the recorded position data of the fingers, hand and arm for the different actions. This data was reduced in the PCA profiles that contained position and time sampling data for the actions. The position variance over time seems to be the primary factor for the observed classification behavior of the ML techniques. It is an open question as to what specific information humans are using in their classification behavior.

### 4.1 Limitations

The dataset used in the training sessions was not completely balanced. For the social/non-social actions, there were 31 social actions and 44 non-social actions. For the grasping/non-grasping actions, there were 34 vs. 41 respectively. One reason for the performance of SVM may be due to this imbalanced dataset. This difference is not large but does deviate from the standard 50/50 proportion for two-class classification. The size of the training datasets might affect the SVM results more than the other ML techniques. We cannot draw clear conclusions about the difference between SVM and the other ML techniques based solely on the original distinctions between grasping/non-grasping and social/non-social.

The methods using nearest neighbors try to classify a gesture based on the training data, which is labeled by the ground truth. The hypothesis of the classification method is that the two classes (grasping vs. non-grasping or social vs. non-social) have distinct kinematic features, and the classification results support this. However, the human ratings did not always align with the ground truth. Thus in some cases the ML algorithms were able to correctly classify the gestures while the humans could not. This suggests that humans use additional information such as prior knowledge other than the position of the light points used by ML techniques to make decisions which is consistent with previous results from (Amoruso and Urgesi, 2016) where they showed that there can be a contextual modulation during action observation and that this modulation is related to motor resonance.

### 4.2 Conclusions

It seems reasonable on the basis of the obtained results that the kinematic information in the profiles is driving the largely successful classification behavior of kNN and LSH-F. We will likely need more gesture instances to improve classification. The current results support previous findings that demonstrate a kinematic basis for perceiving intentions in humans (Ansuini et al., 2014; Sciutti et al., 2015; Cavallo et al., 2016; Becchio et al., 2018).

Movement kinematics (e.g., acceleration, velocity, hand and finger position, change in direction) provide a clear basis for ratings of grasping gestures and social gestures for both humans and ML techniques.

One important issue in the results concerns the more specific information that contributes to the classification distinction between social and non-social gestures given by the kinematic patterns of hand and arm movement. The input to the ML techniques was the recorded position variance of the fingers, hand and arm for the different actions. The position variance over time seems to be the primary factor for the observed classification behavior of the ML techniques. It is an open question as to what specific information humans are using in their classification behavior.

The difference between highly informative movements and less informative movements could play a role in the differences between the ratings of different gestures (Koul et al., 2019). The lack of contextual information (in point-light displays) will create greater dependency on the available kinematic information. The key aspect here then concerns the extent to which the kinematic information is sufficiently informative to drive gesture and intention recognition. In the case of grasping, we originally speculated that the kinematic information was highly informative, and therefore the distinction between grasping and non-grasping for the ML techniques would be more similar to the human ratings than for the social gestures, which did not turn out to be the case. The results from the ML techniques suggest that social gestures are highly informative even when they completely lack any social contextual information because the only visible information is the initial movement of 22 markers. The markers become highly informative when they start to move (Cavallo et al., 2016) and trigger expectations concerning the biological motion associated with different hand gestures.

Our results show a similarity between humans and ML techniques regarding the rating judgments for grasping and social gestures, with the possible exception of SVMs given the current data. One important step in developing this finding in future studies is to begin to analyze the actual predictive value of different phases of social gestures in relation to non-social gestures for both humans and ML techniques. Results from such future studies would provide a better understanding of how humans process the gradual unfolding of movement kinematics and how social robots might be developed to reliably interact with humans.

## Data Availability

The raw data supporting the conclusion of this article will be made available by the authors, without undue reservation.

## Footnotes

1
https://www.his.se/en/research/informatics/interaction-lab/description-of-action-library/

2
https://scikit-learn.org/stable/modules/generated/sklearn.ensemble.RandomForestClassifier.html#sklearn.ensemble.RandomForestClassifier.predict_proba
